# SWIFTCORE: a tool for the context-specific reconstruction of genome-scale metabolic networks

**DOI:** 10.1186/s12859-020-3440-y

**Published:** 2020-04-15

**Authors:** Mojtaba Tefagh, Stephen P. Boyd

**Affiliations:** Information Systems Laboratory, Department of Electrical Engineering, Stanford University, Stanford, US

**Keywords:** Systems biology, Metabolic network analysis, Metabolic network reconstruction, Context-specific models

## Abstract

**Background:**

High-throughput omics technologies have enabled the comprehensive reconstructions of genome-scale metabolic networks for many organisms. However, only a subset of reactions is active in each cell which differs from tissue to tissue or from patient to patient. Reconstructing a subnetwork of the generic metabolic network from a provided set of context-specific active reactions is a demanding computational task.

**Results:**

We propose swiftcc and swiftcore as effective methods for flux consistency checking and the context-specific reconstruction of genome-scale metabolic networks which consistently outperform the previous approaches.

**Conclusions:**

We have derived an approximate greedy algorithm which efficiently scales to increasingly large metabolic networks. swiftcore is freely available for non-commercial use in the GitHub repository at https://mtefagh.github.io/swiftcore/.

## Background

*Constraint-based reconstruction and analysis* (COBRA) is the current state-of-the-art in the genome-scale metabolic network modelling [1]. COBRA methods systematize biochemical constraints into a mathematical framework which synthetic biologists can utilize to quantitatively simulate metabolic pathways in order to answer the relevant biological questions. There is a critical mass of studies that combine these curated high-dimensional models and *in silico* analysis for drug discovery or many other applications [2–4].

Context-specific metabolic networks are extensively studied because of their higher explanatory and predictive power [5–8]. To date, a wide variety of computational methods have been developed to extract context-specific metabolic networks from the available comprehensive genome-scale reconstructions. *Gene inactivity moderated by metabolism and expression* (GIMME) [9] uses quantitative gene expression data and presupposed cellular functions to predict the subset of reactions that a cell uses under particular conditions. *Integrative metabolic analysis tool* (iMAT) [10, 11] integrates tissue-specific gene- and protein-expression data to produce context-specific metabolic networks. *Integrative network inference for tissues* (INIT) [12–14] uses cell type specific proteomic data from the *human proteome atlas* (HPA) to reconstruct tissue-specific metabolic networks. Metaproteomic and taxonomic data have been exploited for a context-specific reconstruction procedure applied to a naphthalene-degrading bacterial community [15]. RegrEx [16, 17] is based on regularized least squares optimization using publicly available RNAseq expression profiles. *Context-specificity assessed by deterministic reaction evaluation* (mCADRE) [18] is also based on gene expression data but evaluates the functional capabilities during model building too. Another approach is to infer the metabolic functionalities of a cell or tissue from transcriptomic data, and then protect these functions during the implementation of context-specific reconstruction [19]. *Cost optimization reaction dependency assessment* (CORDA) [20] relies solely on *flux balance analysis* (FBA), rendering it more computationally efficient.

One approach to this problem is to curate a list of active reactions and develop a framework to find the sparsest subnetwork containing the specified reactions. Although this subnetwork is assumed to be as sparse as possible, we should avoid flux inconsistencies such as when one of the desired reactions is included in the subnetwork, yet it is blocked and cannot carry non-zero flux at the steady-state condition. In this regard, context-specific reconstructions seek to generate minimal consistent subnetworks including a given set of core reactions.

It is known that identifying a consistent subnetwork with the minimum possible size such that it contains some core reactions is an NP-hard problem [21]. To address this issue, approximate greedy algorithms have been developed which either prune the original network [5] or increment the core set [22] recursively to arrive at a consistent subnetwork in between them. Among these competing approaches, FASTCORE [22] exhibits superior performance both in terms of the sparseness of the subnetwork and the computational efficiency.

Let $\mathcal {M} = \{M_{i}\}_{i=1}^{m}$ denote *m* specific metabolites in an organism, and $\mathcal {R} = \{R_{i}\}_{i=1}^{n}$ be the set of *n* reactions involving at least one of these metabolites. Under average physiological conditions, the irreversible reactions $\mathcal {I} \subseteq \mathcal {R}$ are thermodynamically constrained to proceed in the forward direction only, in contrast to the reversible reactions which may proceed in the reverse direction as well.

We call a vector *v* of length *n* a flux distribution if the absolute values of its entries are the rates of the corresponding reactions in $\mathcal {R}$, and the signs of its entries indicate the forward and reverse directions. Unless stated otherwise, all flux distributions are assumed to respect the irreversibility conditions in the sense that *v*_*i*_≥0 for all $R_{i}\in \mathcal {I}$.

We represent the relative quantities of metabolites in each reaction by an associated vector of length *m* and distinguish reactants from products by negative signs. Afterward, we construct the stoichiometric matrix by stacking these vectors for all the reactions in $\mathcal {R}$ as the columns of an *m*×*n* matrix *S*. The mass balance constraint asserts that the concentration of each metabolite is constant throughout the time-scale of interest which is equivalent to say that *Sv*=0 in our notation.

We refer to any solution of *Sv*=0 such that *v*_*i*_≥0 for all $R_{i}\in \mathcal {I}$ as a steady-state flux distribution. We call $R_{i}\in \mathcal {R}$ a blocked reaction if *v*_*i*_=0 for all the steady-state flux distributions, and unblocked otherwise. We call a metabolic network with no blocked reactions a flux consistent metabolic network [23].

In this paper, we present an algorithm that given a flux consistent metabolic network and the subset $\mathcal {C}\subset \mathcal {R}$ of core reactions as input, computes a flux consistent subnetwork $\mathcal {N}\subseteq \mathcal {R}$ such that $\mathcal {C}\subseteq \mathcal {N}$ as output. Ideally, we are interested to find a sparse subnetwork, and accordingly, we search to minimize the size of $\mathcal {N}$.

## Related works

A closely related problem to the context-specific reconstruction is to check the flux consistency of a given metabolic network by detecting the blocked reactions. FASTCC [22] which is based on the same ideas as used for FASTCORE, is currently the fastest algorithm dedicated to this task.

As a simple observation, all the irreversible reactions in $\mathcal {I}$ are unblocked if and only if we can find a flux distribution *v* such that

1 $$ \begin{array}{l} S v = 0 \\ v_{\mathcal{I}} > 0, \end{array}  $$

where $v_{\mathcal {I}} > 0$ is the shorthand of *v*_*i*_>0 for all $R_{i}\in \mathcal {I}$. Assuming that such a flux distribution *v* exists, an arbitrary (possibly reversible) $R_{j}\in \mathcal {R}$ is unblocked if and only if there exists *u* such that

2 $$ \begin{array}{l} S u = 0 \\ u_{j} \neq 0, \end{array}  $$

since if *c*>0 is large enough, then *u*_*j*_+*cv*_*j*_≠0 and *u*+*cv* would be a steady-state flux distribution in which *R*_*j*_ is active.

*Quantitative flux coupling analysis* (QFCA) [24] uses this observation to develop a consistency checking technique which we call SWIFTCC in this paper. However, this is presented as only a preprocessing step in the original paper instead of a separate algorithm. For the sake of completeness, we have also compared SWIFTCC as implemented in the QFCA against FASTCC. Additionally, we have benchmarked FASTCC++ and SWIFTCC++ which are the original algorithms plus the preprocessing step explained in the Appendix. Later on, we extend the ideas of SWIFTCC to develop an analogous method for the context-specific reconstruction problem with the same order of speed-up which SWIFTCC offers.

## Methods

By similar arguments to the previous section, we concluded that a subnetwork $\mathcal {N}$ is flux consistent if and only if

1. there exists *v* in analogy to (1) such that
  $$\begin{array}{l} S v = 0 \\ v_{\mathcal{I}\cap\mathcal{N}} > 0 \\ v_{\mathcal{R}\setminus\mathcal{N}} = 0, \end{array} $$
2. and there exists a set $\{u^{k}\}_{k=1}^{K}$ in analogy to (2) such that
  3 $$ \begin{array}{l} S u^{k} = 0 \\ u_{\mathcal{R}\setminus\mathcal{N}}^{k} = 0, \end{array}  $$
  holds for all 1≤*k*≤*K*. Furthermore, for any $R_{j}\in \mathcal {N}\setminus \mathcal {I}, u^{k}_{j} \neq 0$ holds for at least one 1≤*k*≤*K*.

We start by finding a *v* which is sparse in $\mathcal {R}\setminus \mathcal {C}$ by minimizing the *l*_1_ norm [25]

$$\begin{array}{ll} \text{minimize} & \left\| v_{\mathcal{R}\setminus\mathcal{C}}\right\|_{1} \\ \text{subject to} & S v = 0 \\ & v_{\mathcal{I}\cap\mathcal{C}} > 0 \\ & v_{\mathcal{I}\setminus\mathcal{C}} \geq 0, \end{array} $$

and setting the initial $\mathcal {N}$ to be the non-zero indices of *v*. Consequently, the optimal *v* satisfies 1 for this $\mathcal {N}$.

This homogeneous problem is equivalent to the following *linear program* (LP)

4 $$ \begin{array}{ll} \text{minimize} & \mathbf{1}^{T} w \\ \text{subject to} & S v = 0 \\ & v_{\mathcal{I}\cap\mathcal{C}} \geq \mathbf{1} \\ & v_{\mathcal{I}\setminus\mathcal{C}} \geq 0 \\ & w \geq v_{\mathcal{R}\setminus\mathcal{C}} \\ & w \geq -v_{\mathcal{R}\setminus\mathcal{C}}, \end{array}  $$

by scaling *v* if necessary, so that the nonzero entries of $v_{\mathcal {I}\cap \mathcal {C}}$ are greater than or equal to one.

We define the set $\mathcal {B} = \mathcal {N}\setminus \mathcal {I}$ to be the reactions in $\mathcal {N}$ which have not been verified to be unblocked yet. Whenever we find a *u*_*k*_ which satisfies the conditions of (3), we update $\mathcal {B}$ by removing the reactions *R*_*j*_ for which $u^{k}_{j} \neq 0$, and we also update $\mathcal {N}$ by adding the same reactions to it if they are not already included.

Let $S_{\mathcal {N}}$ denote the matrix consisting of the columns of *S* which correspond to $\mathcal {N}$. We initialize $\{u^{k}\}_{k=1}^{K}$ to be a basis for the set of vectors satisfying (3). Note that, this can be obtained by the *singular value decomposition* (SVD) of $S_{\mathcal {N}}$ since it is a basis for the null space of $S_{\mathcal {N}}$ padded by zeros for the indices corresponding to $\mathcal {R}\setminus \mathcal {N}$. This step is not required and can be skipped to decrease the runtime.

In order to generate the next *u*^*k*^, we consider the solution of

$$\begin{array}{ll} \text{minimize} & x^{T} u_{\mathcal{B}} + \left\|u_{\mathcal{R}\setminus\mathcal{N}}\right\|_{1} \\ \text{subject to} & S u = 0 \\ & \left\|u_{\mathcal{B}}\right\|_{\infty} \leq \mathbf{1}, \end{array} $$

where *x* is a random vector generated from the zero-mean normal distribution with variance *σ*^2^. This problem tries to find a *u* which is sparse in $\mathcal {R}\setminus \mathcal {N}$ by minimizing the *l*_1_ norm, and which is dense in $\mathcal {B}$ by the *l*_*∞*_ norm constraint [26]. The choice of the variance *σ*^2^ manages the trade-off between these two objectives and is set by a simple rule which doubles *σ* whenever $u_{\mathcal {B}}$ is not dense enough, for instance whenever the size of $\mathcal {B}$ is not reduced by more than half. In addition, we have tried several other heuristics, however, SWIFTCORE is robust across a wide range of *σ*.

Finally, this problem is equivalent to the following LP

5 $$ \begin{array}{ll} \text{minimize} & x^{T} u_{\mathcal{B}} + \frac{1}{\sigma}\mathbf{1}^{T} w \\ \text{subject to} & S u = 0 \\ & u_{\mathcal{B}} \leq \mathbf{1} \\ & -u_{\mathcal{B}} \leq \mathbf{1} \\ & u_{\mathcal{R}\setminus\mathcal{N}} \leq w \\ & -u_{\mathcal{R}\setminus\mathcal{N}} \leq w, \end{array}  $$

where *x* is sampled from the standard normal distribution. More generally, $\frac {1}{\sigma }\mathbf {1}$ can be substituted by any positive weight vector *ω* to customize the loss corresponding to each reaction. In the current work, we have only experimented with $\omega = \frac {1}{\sigma }\mathbf {1}$ for different values of *σ*. However, the accompanying package supports any positive weight vector *ω*.

Subsequently, we keep iterating over *k* until no reaction remains in $\mathcal {B}$. Therefore, we will eventually arrive at a set $\{u^{k}\}_{k=1}^{K}$ which satisfies 2 for the final $\mathcal {N}$, thus together with the prior *v*, they imply that $\mathcal {N}$ is a flux consistent subnetwork. Note that, we can also impose lower and upper bound constraints on the feasible flux distributions by adding the corresponding inequalities to (4) and (5) for the general case.

Altogether, this algorithm is based on linear programming, and hence, SWIFTCORE is ultra-fast in comparison to nonlinear methods such as the *model-building algorithm* (MBA) [5] which are orders of magnitude slower. Focusing on computational efficiency, the only real contender is the FASTCORE algorithm which is again based on linear programming but is still several times slower, mainly due to flipping the signs of the columns of the stoichiometric matrix that correspond to the reversible reactions. We refer the interested reader to the associated paper [22] for more details on this technique which we have avoided in SWIFTCORE in the way explained below.

Whether it is a binary variable in a *mixed-integer linear program* (MILP) corresponding to the direction of a reversible reaction or two separate iterations over the forward and reverse directions like in the FASTCORE, a common way of dealing with reversible reactions is to iterate over instances of the metabolic network with a predetermined direction for that reversible reactions. Instead of determining a direction for the reversible reactions, signs of the different entries of *x* merely encourage one of the two possible directions in a “soft” manner instead of a “hard” constraint. The objective function of (5) is minimized when most of the entries of $u_{\mathcal {B}}$ have the opposite sign to the corresponding entries of *x*. However, it may not be the case for some entries of the solution, especially entries with small absolute values. In this sense, even though the sampled *x* assigns random preferences to the direction of every reversible reaction in $\mathcal {B}$, the optimal *u* might have different signs in a few entries. As a result, the search for a sparse *u* is conducted over a much larger feasible set, and $\mathcal {B}$ vanishes in fewer iterations.

As a side note, SWIFTCORE also preprocesses the original metabolic network by merging the pairs of reactions which are fully coupled to each other by a metabolite with no other adjacent reaction (*cf.*, Figure 1 in [27]). However, this optional subroutine can be skipped safely and only affects the runtime. Besides, we can repeat the whole algorithm with the same core reactions $\mathcal {C}$ but replace the original metabolic network by $\mathcal {N}$ to further shrink the subnetwork until its size is no longer reduced.

## Implementation

We have implemented both SWIFTCC and SWIFTCORE in an open-source package freely available for academic use on GitHub. It is written in MATLAB^®^ with zero dependencies, though it supports Gurobi™ or CPLEX™ optimizer for improved performance. Additionally, if any other LP solver has been set up for the COBRA toolbox v3.0 [28], it can use that as well by calling the LP solve function of COBRA.

The partitioning preprocessing step described in the Appendix is also included in this package. Despite the fact that in our simulations it does not improve the performance directly, we suggest exploiting its potential to develop parallel consistency checking methods as the partitioned subnetworks can be analyzed independently. We did not investigate how much this might improve the efficiency of either SWIFTCC or FASTCC none of which can readily be parallelized in an obvious way and this direction is left for future research.

Last but not least, the benchmark codes to reproduce all the figures in this article are also publicly available in the same GitHub repository.

## Results and discussion

To assess the performance of SWIFTCORE, we have benchmarked both SWIFTCORE and FASTCORE on the flux consistent part of the Recon3D model [29] with randomly selected core sets of varying sizes over the range of 1 to the number of reactions *n*=10600. All simulations were performed on a desktop PC with AMD Ryzen™7 1800X eight-core processor and 16 GB of memory, and the CPLEX™ optimizer was employed as the LP solver. Furthermore, in the published benchmark code we double check if the reconstructed subnetwork is flux consistent and contains the core reactions and SWIFTCORE always passes these sanity checks.

**Fig. 1:**
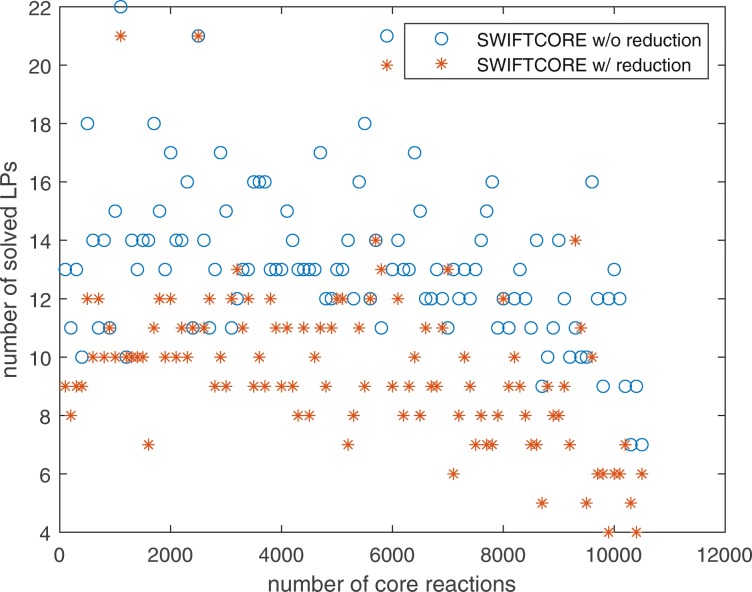
SWIFTCORE requires to solve at most 22 LPs on Recon3D with *n*=10600

**Fig. 2:**
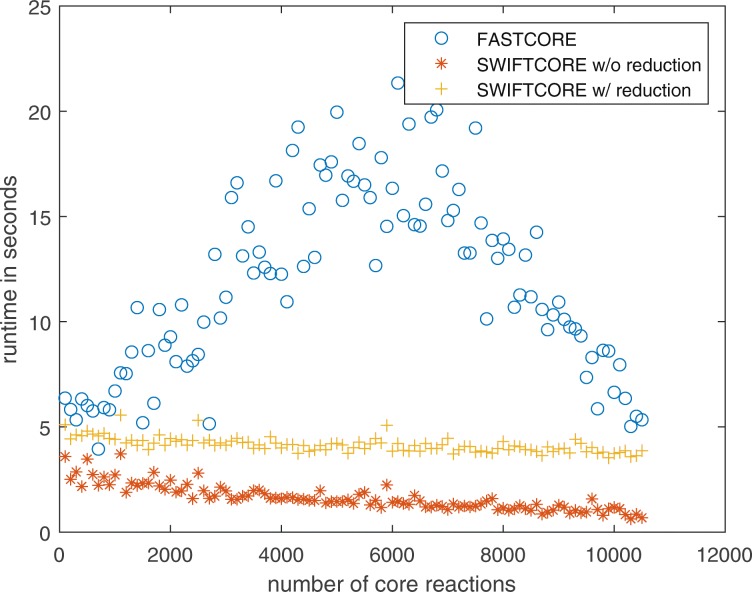
SWIFTCORE is 6× faster than FASTCORE on average over these 105 iterations of varying sizes

In Fig. 1, the difference between the two versions of the algorithm is that in the SWIFTCORE with reduction version we have included the SVD and full coupling reduction techniques described before in contrast to the other vanilla version which excludes them. In spite of the fact that the vanilla version solves more LP problems, it is actually more efficient nonetheless (see Fig. 2).

**Fig. 3:**
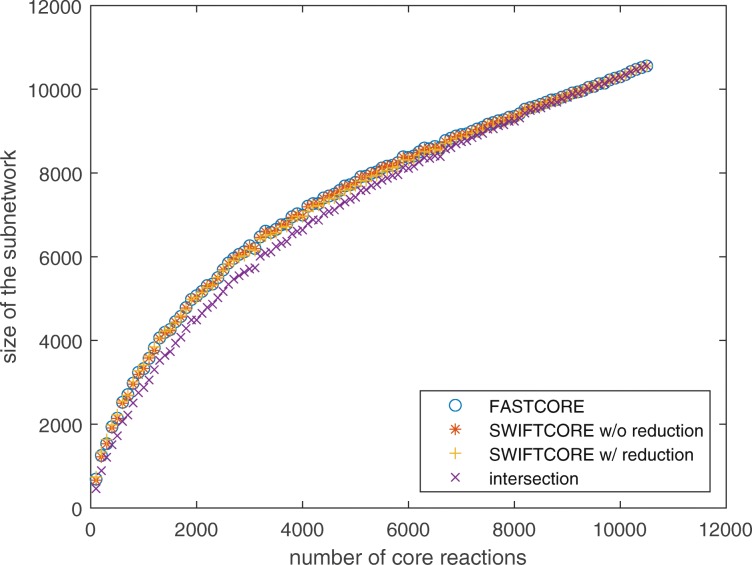
Benchmark of SWIFTCORE against FASTCORE on Recon3D

Figure 3 shows that the sparsity of the outputs of all three algorithms is nearly identical. Moreover, the intersection of the three solution subnetworks usually accounts for about 95% of the reactions which means the subnetworks are almost the same.

**Fig. 4:**
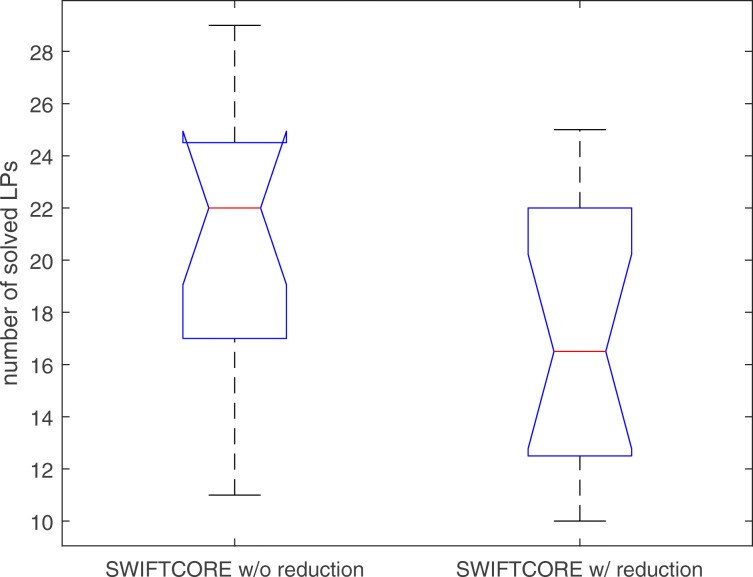
The number of solved LPs changes proportional to the logarithm of the scaling factor

**Fig. 5:**
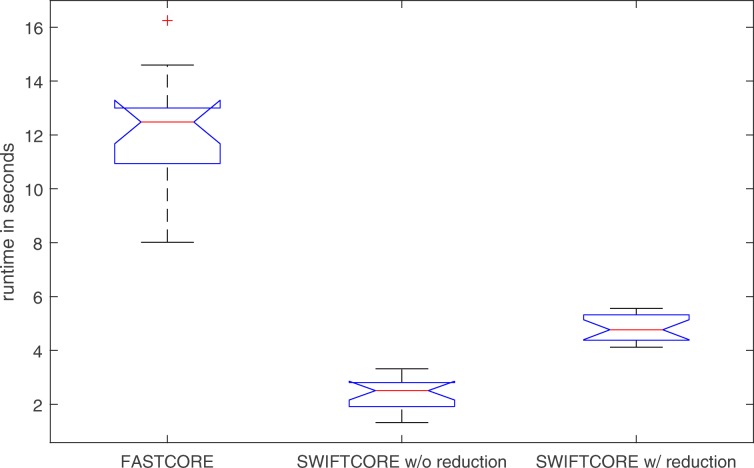
The variation in runtime is relatively smaller for SWIFTCORE compared to FASTCORE

**Fig. 6:**
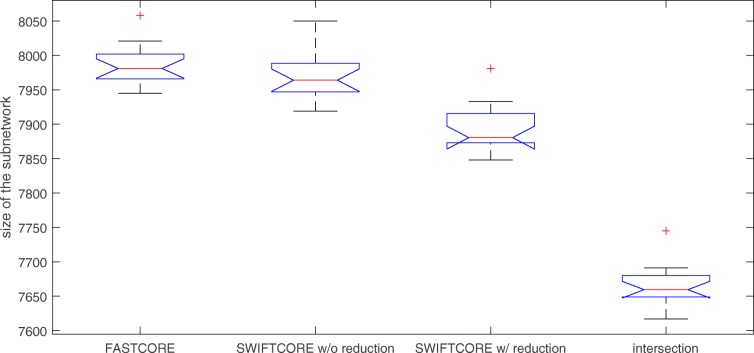
The difference in sparsity is less than 1% for all algorithms

Regarding the impact of the weights, Figs. 4, 5, and 6 demonstrate that the number of solved LPs, the runtime, and the sparsity of the output subnetworks is pretty robust with respect to the variations of weights. In these figures, we randomly sample half of the reactions in the network and run both algorithms with the weight vectors 2^*k*^**1** for *k*=0,1,…,15. Note that the magnitude of the largest weight vector is 32768 times the magnitude of the smallest one yet again, as claimed before, SWIFTCORE is robust to the choice of *σ*.

**Fig. 7:**
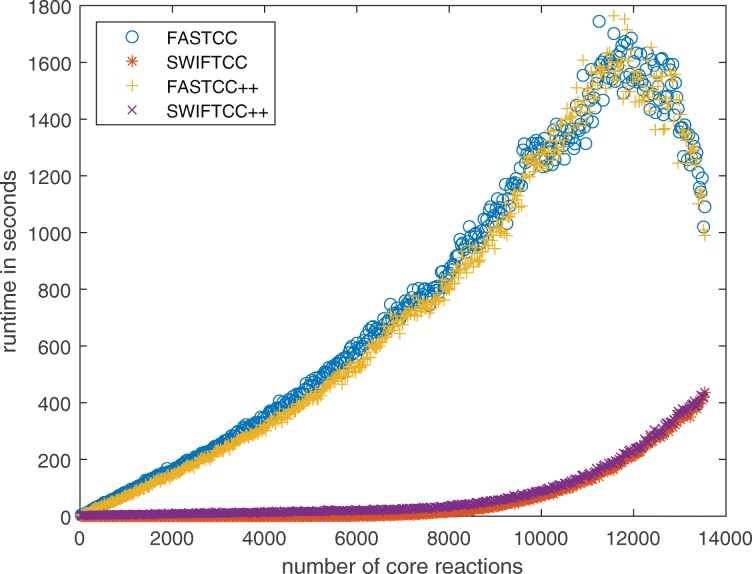
SWIFTCC is more than 12× faster than FASTCC on average over these 467 iterations of varying sizes

**Fig. 8:**
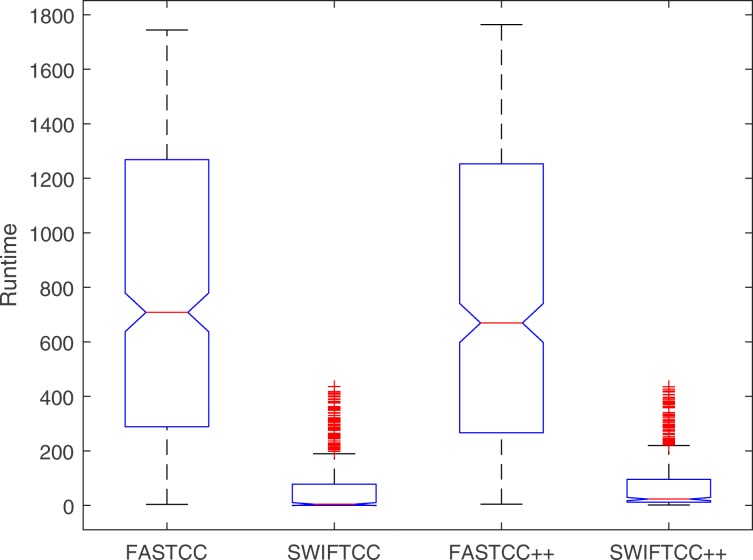
FASTCC++ is only 4% faster than FASTCC on average

In the end, we have also benchmarked SWIFTCC against FASTCC on the flux inconsistent version of the Recon3D model and its randomly selected subnetworks. In Figs. 7 and 8, SWIFTCC’s runtime is 8% of FASTCC’s runtime on Recon3D model averaged over sampled subnetworks of different sizes from 1 to *n*=13543.

**Fig. 9:**
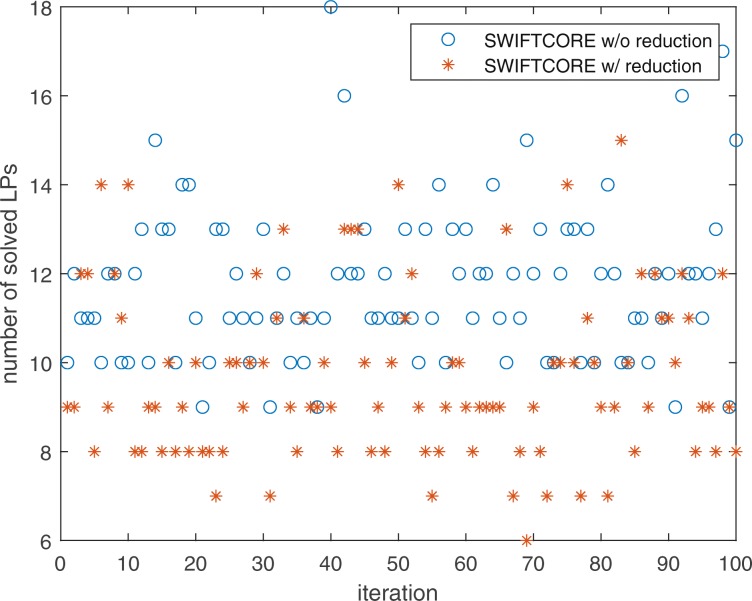
SWIFTCORE requires to solve at most 18 LPs for the hepatocyte-specific reconstruction

**Fig. 10:**
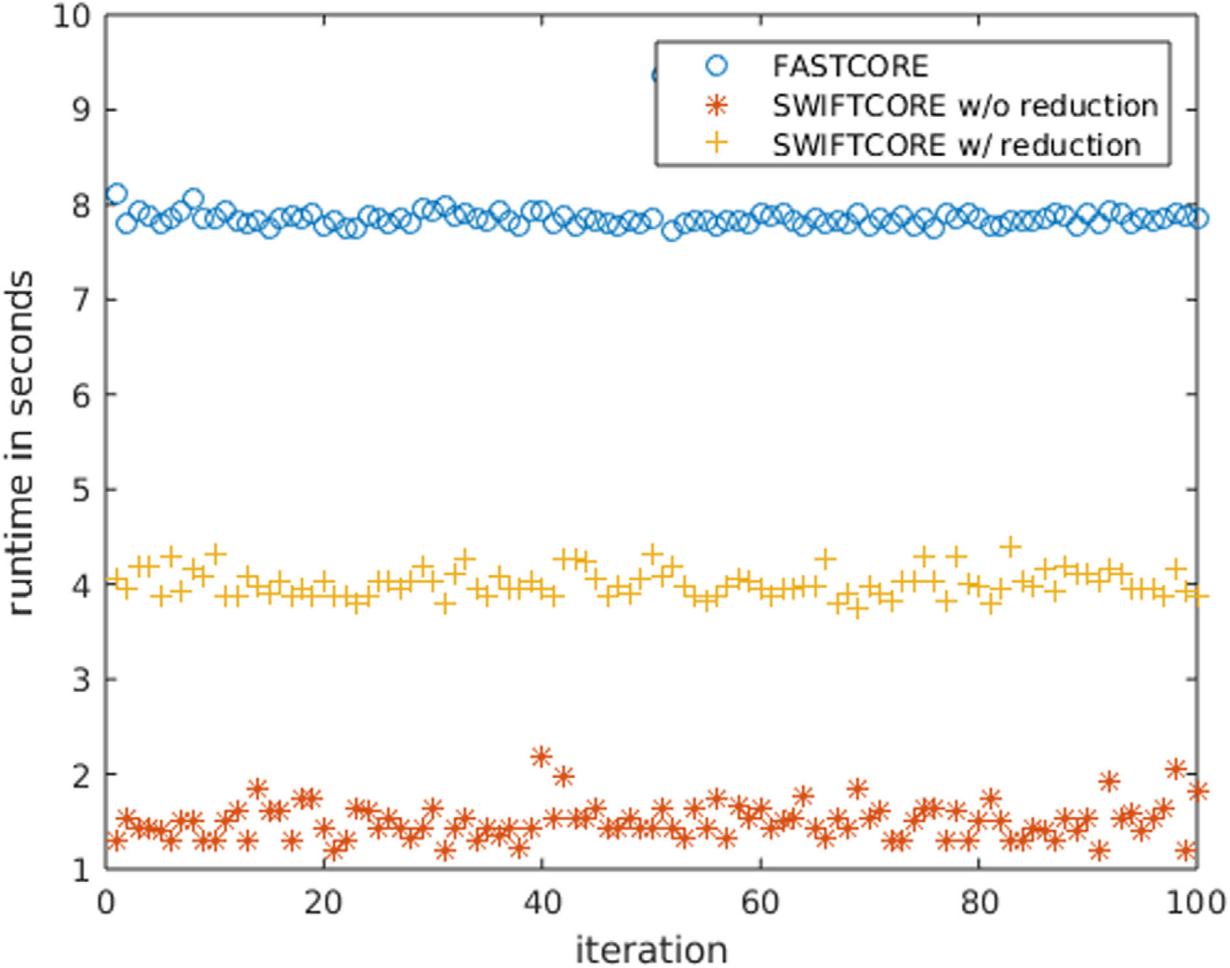
SWIFTCORE is more than 5× faster than FASTCORE on average over these 100 iterations

In a second setting, we evaluated both algorithms by the reconstruction of a liver model previously studied in the original MBA and FASTCORE articles [5, 22]. All simulations were performed on a laptop with Intel^Ⓒ^ Core™i7-5500U CPU @ 2.40GHz ×2 and 16 GB of memory, and the Gurobi™ optimizer was employed as the LP solver. Figures 9 and 10 are consistent with our previous findings.

**Fig. 11:**
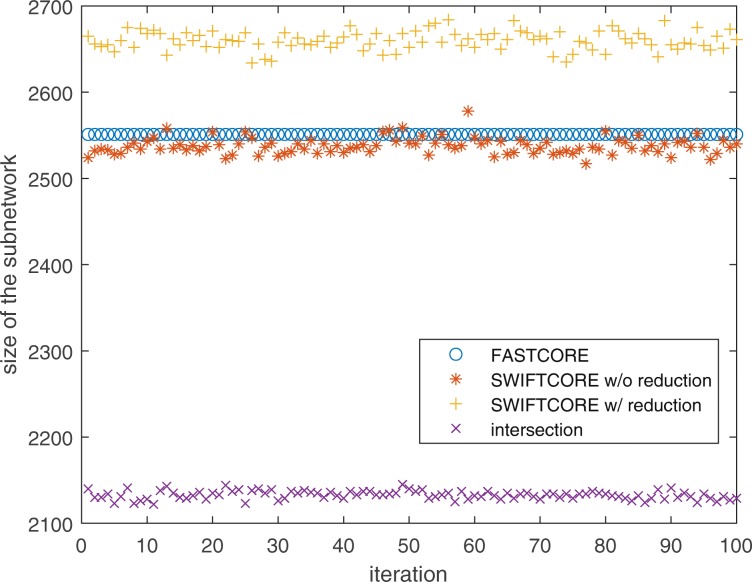
SWIFTCORE with reduction is 4% less sparse and without reduction is 1% sparser than FASTCORE

**Fig. 12:**
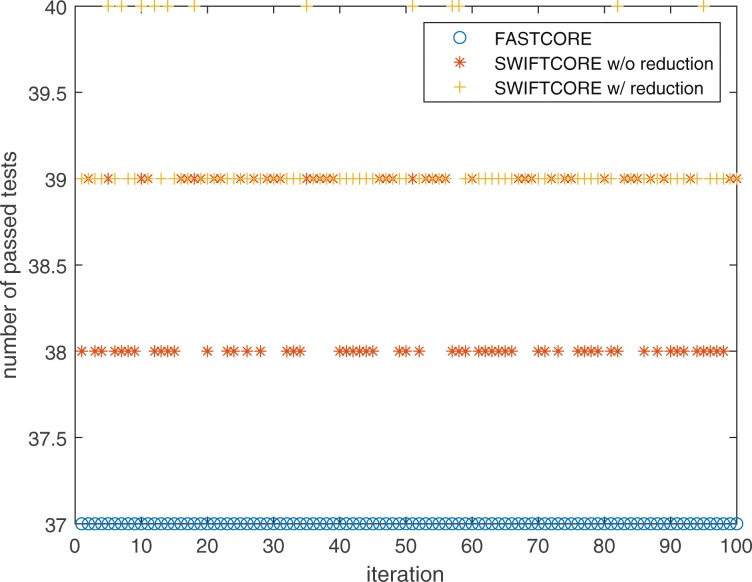
SWIFTCORE with reduction passes 2.12 and without reduction passes 1.44 more tasks than FASTCORE on average

Since FASTCORE is a deterministic algorithm, we see a corresponding flat line in Fig. 11 because of the same hepatocyte-specific core set used in every iteration. On the other hand, the randomly selected *x* in (5) makes SWIFTCORE a stochastic algorithm which samples the space of alternative optimal subnetworks. It has been proposed that a careful balance between model sparsity and metabolic functionality helps in reducing the ambiguity of context-specific metabolic network predictions [17, 30]. In order to do so, we have tested all reconstructions by the list of data-inferred metabolic tasks recently published in [19]. As we can see in Fig. 12, both versions of the SWIFTCORE algorithm sometimes pass an additional task which can be a guidelines to obtain a more biologically relevant reconstruction from the sampled alternative optima.

## Conclusions

Here, we have presented SWIFTCC and SWIFTCORE and one can easily implement a corresponding SWIFTGAPFILL by substituting the subroutine calls to FASTCC and FASTCORE in the FASTGAPFILL [31]. We have demonstrated through a real-world genome-scale model that the SWIFTCORE family improves the efficiency of metabolic network reconstruction significantly.

## Availability and requirements

**Project name:**SWIFTCORE

**Project home page:** https://mtefagh.github.io/swiftcore/

**Operating system(s):** Platform independent

**Programming language:** MATLAB^®^

**Other requirements:** The COBRA and Bioinformatics toolboxes for MATLAB; use of Gurobi™ or CPLEX™ is optional for improved performance (free academic licenses are available for both)

**License:**SWIFTCORE is distributed under the GNU GPL v3.0.

**Any restrictions to use by non-academics:** licence needed

## Appendix

To the end of this Appendix, without loss of generality we assume that all the reactions are irreversible, i.e., $\mathcal {I} = \mathcal {R}$. If this is not the case, we can replace each reversible reaction by a pair of irreversible reactions corresponding to its forward and reverse directions.

We define the skeleton digraph of a metabolic network as $G = (\mathcal {M}, \mathcal {A})$ where $\mathcal {M}$ is the set of metabolites and $\mathcal {A}$ is the set of ordered pairs of metabolites (*M*_*i*_,*M*_*j*_) for which there exists a reaction $R_{k}\in \mathcal {R}$ whose reactants and products include *M*_*i*_ and *M*_*j*_, respectively.

We define the mapping $f:\mathcal {R} \longrightarrow \mathcal {P}(\mathcal {A})$ by

$$f(R_{k}) = \{(M_{i},M_{j}) \mid S_{ik} < 0, S_{jk} > 0 \}, $$

where $\mathcal {P}(\mathcal {A})$ denotes the power set (the set of all the subsets) of $\mathcal {A}$. Moreover, we define its extension $\tilde {f}:\mathcal {P}(\mathcal {R}) \longrightarrow \mathcal {P}(\mathcal {A})$ by

$$\tilde{f}(\tilde{\mathcal{R}}) = \bigcup_{R_{k}\in\tilde{\mathcal{R}}}f(R_{k}). $$

Note that, $\tilde {f}(\mathcal {R})=\mathcal {A}$. Intuitively, $\tilde {f}$ of a subset of reactions $\tilde {\mathcal {R}} \subseteq \mathcal {R}$ is the corresponding directed arcs in $\mathcal {A}$ by breaking any hyperarcs in $\tilde {\mathcal {R}}$ into directed arcs.

Let $\mathcal {A} = \{A_{1}, A_{2}, \ldots, A_{a}\}$, and *S*_*k*_ denote the *k*th column of *S*. Suppose that **1**^*T*^*S*=0. Then for any $R_{k}\in \mathcal {R}$ we can define

$$C_{k} = -\sum_{S_{ik} < 0}S_{ik} = \sum_{S_{jk} > 0}S_{jk} = \frac{1}{2}\sum_{l=1}^{m} |S_{lk}|, $$

and we have that

$$\begin{aligned} S_{k} &= \sum_{S_{ik} < 0} S_{ik}e_{i} + \sum_{S_{jk} > 0} S_{jk}e_{j} \\ &= \sum_{S_{ik} < 0} \sum_{S_{jk} > 0} \frac{S_{ik} S_{jk}}{C_{k}} e_{i} - \sum_{S_{jk} > 0} \sum_{S_{ik} < 0} \frac{S_{ik} S_{jk}}{C_{k}} e_{j} \\ &= \sum_{S_{ik} < 0, S_{jk} > 0} \frac{-S_{ik} S_{jk}}{C_{k}} (e_{j}-e_{i}). \end{aligned} $$

Therefore,

$$S = \partial F, $$

where *∂* is the incidence matrix of *G* and *F* is the nonnegative *l*×*n* matrix whose entries are

$$f_{lk} = \frac{-S_{ik} S_{jk}}{C_{k}}, $$

if *A*_*l*_=(*M*_*i*_,*M*_*j*_)∈*f*(*R*_*k*_) and *f*_*lk*_=0 if *A*_*l*_∉*f*(*R*_*k*_).

The support of a vector *v*∈**R**^*n*^, denoted by supp(*v*), is defined to be the set of its nonzero indices. With a slight abuse of notation, this is also used to show the set of reactions in $\mathcal {R}$ which are active in a flux distribution, i.e.,

$$\text{supp}(v) = \{R_{i}\in\mathcal{R} \mid v_{i}\neq 0\}. $$

Suppose that **1**^*T*^*S*=0. We claim that $\tilde {f}(\text {supp}(v))$ is a strongly connected subgraph of *G* for any steady-state flux distribution *v*. The sketch of the proof is to first note that

$$\tilde{f}(\text{supp}(v)) = \bigcup_{v_{k}\neq 0}f(R_{k}) = \bigcup_{v_{k}\neq 0}\text{supp}(F_{k}) = \text{supp}(Fv) $$

but then *∂*(*Fv*)=*Sv*=0 and hence supp(*Fv*) is strongly connected and so is $\tilde {f}(\text {supp}(v))$.

This observation gives rise to a preprocessing step for any flux consistency checking algorithm by partitioning *G* into its strongly connected components, and any reaction which is not inside a strongly connected component would be blocked. Then the flux consistency checking algorithm is called on each one of them to get the rest of blocked reactions. It only remains to show that the assumption **1**^*T*^*S*=0 holds for a broad class of metabolic networks with a slight modification to internalize their boundary reactions but without changing their steady-state flux distributions.

The boundary reactions $\mathcal {R}_{B}\subseteq \mathcal {R}$, as the name suggests, lie on the boundary of a given metabolic network, e.g., exchange reactions with extracellular metabolites, and all the reactants utilized or all the products formed by these reactions are external, i.e., their corresponding stoichiometric vectors are either nonnegative or nonpositive. Consequently,

$$\tilde{f}(\mathcal{R}_{B})=\emptyset. $$

On the other hand, internal reactions $\mathcal {R}_{I}$ are the subset of $\mathcal {R}$ which only comprise internal metabolites in $\mathcal {M}$, hence the name internal. Next, we will see that this distinction is necessary for the stoichiometric consistency analysis where we restrict our attention to the internal reactions. Otherwise, the missing information on extracellular metabolites can be misinterpreted as stoichiometric inconsistency in the metabolic network.

Let *S*_*I*_ denote the submatrix of *S* restricted to the columns indexed by $\mathcal {R}_{I}$, and *w*∈**R**^*m*^ denote the positive vector of the molecular masses of $\mathcal {M}$. By the law of mass conservation in a stoichiometrically consistent metabolic network, the sum of molecular masses of $\mathcal {M}$ weighted by their associated stoichiometric coefficients in any arbitrary $R_{i} \in \mathcal {R}_{I}$ must be equal to zero. Therefore, any *w*>0 which fulfils the mass conservation law is assumed to satisfy *w*^*T*^*S*_*I*_=0 by our earlier convention. A metabolic network is called stoichiometrically consistent if there exists at least one molecular mass vector *w* such that

6 $$ \begin{array}{l} S_{I}^{T} w = 0 \\ w > 0, \end{array}  $$

and stoichiometrically inconsistent otherwise [32]. We note that, determining whether a metabolic network is stoichiometrically consistent or not by (6) is the same feasibility problem as (1) by replacing *S* and $\mathcal {I}$ with $S_{I}^{T}$ and $\mathcal {M}$.

Consider the following stoichiometric matrix

$$S' = \left[ \begin{array}{c} W S\\ -w^{T} S \end{array} \right] $$

where *w*>0 is an arbitrary molecular mass vector and *W* is the diagonal matrix whose diagonal entries are *w*. We associate the additional row with a fictitious extracellular metabolite *M*_*m*+1_, and because of *w*^*T*^*S*_*I*_=0, the stoichiometry of the internal reactions do not involve this newly added metabolite. Since the last row of *S*^′^ is a linear combination of the other rows, we have *S*^′^*v*=0⇔*Sv*=0. Therefore, we can replace *S* by *S*^′^ and the set of the steady-state flux distributions does not change. Furthermore, if *S*_*i*_ is either nonnegative or nonpositive, then the *i*th entry of −*w*^*T*^*S* has the other sign and hence with respect to *S*^′^ all reactions are internal, i.e., $S^{\prime }_{I} = S'$. The boundary reactions which were previously defined to have either nonnegative or nonpositive stoichiometric vectors correspond to the reactions which involve the fictitious metabolite *M*_*m*+1_ in this new setting. Eventually,

$$\mathbf{1}^{T} S' = \mathbf{1}^{T} \left[ \begin{array}{c} W S\\ -w^{T} S \end{array} \right] = \mathbf{1}^{T} W S - w^{T} S = 0. $$

As a final remark, we recall that in order to construct *G* and *f* we only need to know the signs of the elements of *S*^′^, thus this can be done without computing *w* explicitly. Even when we do not know whether the metabolic network is stoichiometrically consistent or not, we can still construct *G*, and if it is not strongly connected, we conclude that the metabolic network has either stoichiometric or flux inconsistencies.

## Data Availability

The Recon3D model is publicly available at http://vmh.life.

## Abbreviations

COBRA: Constraint-based reconstruction and analysis
GIMME: Gene inactivity moderated by metabolism and expression
iMAT: Integrative metabolic analysis tool
INIT: Integrative network inference for tissues
HPA: Human proteome atlas
mCADRE: Context-specificity assessed by deterministic reaction evaluation
CORDA: Cost optimization reaction dependency assessment
FBA: Flux balance analysis
QFCA: Quantitative flux coupling analysis
LP: Linear program
SVD: Singular value decomposition
MBA: Model-building algorithm
MILP: Mixed-integer linear program
